# Human Activity Recognition for AI-Enabled Healthcare Using Low-Resolution Infrared Sensor Data

**DOI:** 10.3390/s23010478

**Published:** 2023-01-02

**Authors:** Yordanka Karayaneva, Sara Sharifzadeh, Yanguo Jing, Bo Tan

**Affiliations:** 1 School of Computing, Engineering and Digital Technologies, Teesside University, Middlesbrough TS1 3BX, UK; 2 Faculty of Science and Engineering, Swansea University, Swansea SA2 8PP, UK; 3 Faculty of Business, Computing and Digital Industries, Leeds Trinity University, Leeds LS18 5HD, UK; 4 Faculty of Information Technology and Communication Science, Tampere University, 33100 Tampere, Finland

**Keywords:** human activity recognition (HAR), infrared sensors, noise reduction, feature extraction, classification, AI-enabled healthcare

## Abstract

This paper explores the feasibility of using low-resolution infrared (LRIR) image streams for human activity recognition (HAR) with potential application in e-healthcare. Two datasets based on synchronized multichannel LRIR sensors systems are considered for a comprehensive study about optimal data acquisition. A novel noise reduction technique is proposed for alleviating the effects of horizontal and vertical periodic noise in the 2D spatiotemporal activity profiles created by vectorizing and concatenating the LRIR frames. Two main analysis strategies are explored for HAR, including (1) manual feature extraction using texture-based and orthogonal-transformation-based techniques, followed by classification using support vector machine (SVM), random forest (RF), k-nearest neighbor (k-NN), and logistic regression (LR), and (2) deep neural network (DNN) strategy based on a convolutional long short-term memory (LSTM). The proposed periodic noise reduction technique showcases an increase of up to 14.15% using different models. In addition, for the first time, the optimum number of sensors, sensor layout, and distance to subjects are studied, indicating the optimum results based on a single side sensor at a close distance. Reasonable accuracies are achieved in the case of sensor displacement and robustness in detection of multiple subjects. Furthermore, the models show suitability for data collected in different environments.

## 1. Introduction

Interpretation of human activity for long-term health condition monitoring has become an emerging research topic due to the deployment of smart sensors in residential and caring house environments. This technology allows arising awareness about human wellbeing [1]. The impact of the sensor data can reveal the nature of an activity and hence can indicate the physical and mental condition of subjects in a healthcare context [2].

The important requirements of such systems are (1) accuracy and reliability, and (2) convenience in use and preserving user privacy. While achieving the former requires designing advanced smart HAR algorithms, the latter can be addressed by using nonwearable, nonintrusive sensing technologies.

There are different sensing technologies used for HAR. The fast-evolving microelectromechanical systems (MEMS) has enabled miniaturizing acoustics, radio, and optical sensing components. There are various commodity sensors available in the form of installed or wearables for daily activity monitoring. Typical examples include high-definition camera and depth sensor for detailed human body and facial feature capturing [3], Doppler radars for modeling the dynamics patterns in daily activities, vital sign detecting [4], and wearable accelerometers/gyroscopes for motion and orientation capturing [5]. Nevertheless, the application of vision-based devices for activity recognition is often constrained by privacy concern and low user acceptance [6]. Although powerful, Doppler radars are only sensitive to dynamic movement and cannot detect static poses [7]. Additionally, due to the high kinematics complexity, inter body parts scattering and other RF signal interferences, the Doppler signatures of the moving human body are complicated for subsequent model performance. In terms of wearable sensors, the limited battery life causes issues regarding the need to monitor individuals at all times [8]. In addition, these sensors can be regarded as intrusive, which affects their user acceptance [9]. Infrared sensors are often cheap, small, and easy-to-use devices, which, on the other hand, also come with their disadvantages. The disadvantages mainly include relatively short distance range and sensitivity to background noise; however, they have been proven to achieve robust accuracy for recognition of various daily activities while preserving the subject’s privacy [10].

**Figure 1:**
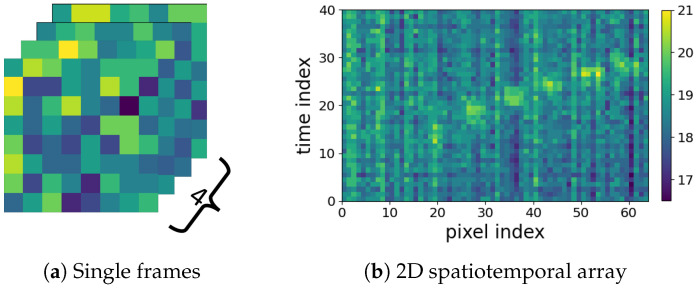
(**a**) Examples of 8×8 pixel frames for the walking diagonally activity, and (**b**) the vectorized spatiotemporal 2D array over 40 frames and 64 pixels.

In this paper, we pay attention to the LRIR array, which is a new type of sensor, providing thermal pixels of a subject’s silhouette [11]. In Figure 1a, four LRIR data frames are shown. Each frame consists of 8×8 thermal pixels. Due to low resolution and lack of local dependencies of pixels, for analyzing the time series of low-resolution frames, they can be vectorized and concatenated over time to form 2D spatiotemporal maps. A total of 40 frames are used, as shown in Figure 1b, which is further explained in Section 3.1.

Compared with PIRs, which only output the binary indicator, the LRIR thermal pixels deliver richer subject information, suitable for activity modeling, but far from personal identification. Thus, the LRIR is potentially a candidate for nonintrusive activity detection and monitoring without the privacy concern in healthcare contexts. Reviewing the literature shows that the use of LRIR has been investigated for fall detection, presence or occupation estimation, motion and proxemics tracking, and activity recognition in different indoor environments [11,12,13,14,15,16,17,18]. This comprehensive review of the current literature allowed us to identify the limitations of the current solutions that impede the widespread use of this novel sensor technology.

One of the main limitations of the existing studies is the lack of detailed understanding about the impact of sensor layout and perspective for optimal deployment of LRIR sensors for different care solutions. The majority of current studies employed a single ceiling-mounted (e.g., [11,12,13,14,15]) or front-mounted sensor [18]. Although a combination of front and side sensors were used in [16], the study lacks comparison of different sensors’ performances. Despite the efforts of the previous literature, there are some unknown factors about the optimal use of LRIR sensors, such as the optimum sensor layout, e.g., distance to subjects, and viewpoint. Additionally, achievable recognition rate, noise removal, and tolerance to changes in sensor layout, for example, due to sensor displacement, still require further research.

Another point is related to identifying the most effective strategies for time series analysis of the IR frames. In some works, the analysis of sequence of frames is employed based on feature extraction followed by classification, while another group of methods are based on time series analysis using DNN methods such as CNN LSTM. Feature extraction is employed for dimensionality reduction purpose in most studies. For example, physical feature extraction was proposed in [11,12,13,14]. Spatiotemporal discrete cosine transform (DCT) for feature extraction was used in [16]. Hosono et al. [18] used a thermo-spatial histogram for feature extraction. In terms of classification of features, different methods, for instance, support vector machine (SVM) [11,14] and k-nearest neighbor (k-NN) [12], are employed. Recently, k-NN, SVM, extreme learning machine (ELM), and convolutional neural networks (CNN) methods have been compared with an LSTM model in [17]. The overall results in that work indicated the superiority of the deep learning model based on LSTM compared to the traditional methods and deep CNN. However, this result cannot be seen as a general conclusion since only a limited dataset was used, including only four activities performed in an empty scene. Besides that, the feature extraction methods were not described. In another study, Bayesian filter was applied for human tracking [15]. In addition, background subtraction for HAR was employed in [14,15,16]. Overall, a holistic comparison of the two main analysis strategies, including feature extraction and classification as well as DNN methods, on different datasets including variety of activities and subjects in more realistic indoor environments is required.

There is a third concern regarding developing an appropriate noise removal preprocessing strategy. Reviewing the literature shows that there are limited studies on noise characteristics and noise reduction techniques for LRIR data. Most recently, the effect of temperature variations was addressed by filtering the LRIR frame pixels using a combination of the J-filter noise reduction method and the Butterworth filter [17]. In another work, Kalman filtering (KF) was used for Gaussian noise reduction [13]. While KFs have shown suitability for dynamic environments, the defined model is expected to ideally correspond to a real-world scenario. Besides the thermal variations causing noise on individual pixels, depending on the way that the sequence of frames are considered in an analysis pipeline, other types of noise might influence the HAR results. For example, in this work, the 2D spatiotemporal maps shown in Figure 1b are considered for HAR. As can be seen, some periodic vertical and horizontal noise appears in the vectorized 2D spatiotemporal profiles. In this paper, a novel noise removal strategy is introduced to address this type of noise as, so far, no effective noise removal strategy has been developed to alleviate this type of periodic noise.

In order to address the reviewed issues for the use of LRIR sensors for HAR, a holistic study is conducted in this paper. In the following, the main contributions are highlighted:

- **LRIR datasets for community:** For the first time, our synchronized multichannel LRIR dataset, referred to as *Coventry-2018* [19], is utilized as the main dataset in this paper for activity recognition. In addition, another existing dataset, the *Infra-ADL2018*, is used in order to verify models based on two different datasets. These are the first two LRIR datasets that include multiple view angles, single, and multiple subjects in the scene. The datasets will help the researchers in the community to identify the optimum experimental settings in terms of the number of required sensors for highest accuracy, the optimum sensor position, noise removal, model generalization, and sensitivity to sensor layouts.
- **Comprehensive comparison and verification of main analysis strategies:** Two main groups of analysis strategies are considered: (1) the 2D spatiotemporal maps shown in Figure 1b are used for applying different feature extraction strategies to achieve deep insight about the new LRIR data. These include (i) orthogonal transformation based on the singular-value decomposition (SVD) and the Fisher’s canonical variables, (ii) two texture feature extraction techniques, including spectral domain analysis using 2D DCT and the gray-level co-occurrence matrix (GLCM). Then, the extracted features are fed to a selected group of classifiers including SVM, k-NN, random forest (RF), and logistic regression (LR) for activity recognition. (2) The series of 8×8 LRIR frames are used as video streams to train a deep convolutional LSTM model for activity detection.
- **Novel periodic noise reduction technique:** To alleviate the horizontal and vertical periodic noise in 2D spatiotemporal maps shown in Figure 1b, a new supervised noise removal algorithm based on Fourier transform is proposed to improve the quality of 2D profiles before feature extraction and activity classification. To the best of our knowledge, no previous research addressed the periodic noise issue for such 2D spatiotemporal maps.
- **Model sensitivity and generalization interpretation:** Leveraging rich sensor settings during data collection, including multiple sensors and layouts, comprehensive interpretations are derived. This includes the following: (1) Feature robustness and model sensitivity and generalization against different layout properties, such as geometry size, prospects, and environmental factors, as well as the recommended optimal room setup and sensor subset; (2) model sensitivity against the number and diversity of the subjects under test, even unseen subjects.

The rest of the paper is organized as follows: Section 2 covers data collection and description. The analysis methods are described in Section 3, and the results are presented in Section 4. Finally, the discussion and conclusion are given in Section 5 and Section 6, respectively.

## 2. Data Collection and Data Description

In this paper, two datasets, named *Coventry-2018* and *Infra-ADL2018* [16], are considered. In this section, the multichannel LRIR sensor system design, experiment layout, and other settings for acquisition of *Coventry-2018* dataset are described, and then, the dataset content in terms of activities and subjects is presented in detail. In addition, the *Infra-ADL2018* dataset captured based on multiple LRIR sensors, in Bristol Robotic Lab, with different subjects and activities is described.

### 2.1. Coventry-2018 Dataset Collection and Description

#### 2.1.1. Sensor and Processing System Design

The *Coventry-2018* dataset was collected in the Faculty of Engineering, Environment and Computing, Coventry University. The Panasonic^®^ Grid-EYE sensor (AMG8833) is used as an instance of LRIR data. Grid-EYE represents the front-view (roughly 60°) scene by an 8×8 thermal pixel array, which is named as *frame* in this work. The multichannel LRIR sensor system consists of three AMG8833 evaluation boards. They are connected to a host PC via universal asynchronous receiver–transmitter (UART) interfaces. Three LRIR data streams are synchronized and timestamped with up to 10 frames per second (FPS) per channel, and saved in a *.csv* format. The 10 FPS is chosen for (1) having appropriate acquisition rate to capture frames and having detailed states transition records during gesture cycle; (2) reserving enough time for sensor reaction to the changes in temperature and obtaining accurate thermal reading. The configuration of the AMG8833 evaluation boards and controlling of the data streams are coordinated by National Instruments LabVIEW^®^.

#### 2.1.2. Sensors Layout

**Figure 2:**
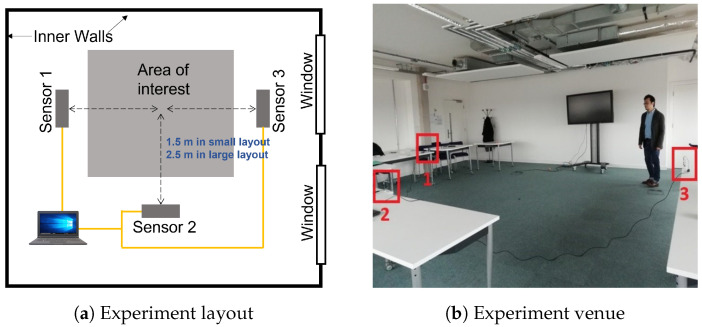
Sensor layouts and experiment scenario. (**a**) Small and large experiment layouts; (**b**) a real experiment scenario with the three sensors.

Two layouts for positioning the sensors in the experiment scene are considered in this work. They are *small layout* and *large layout*. The geometry details are shown in blue color in Figure 2a. In the small layout, three Grid-EYE sensors are placed equally 1.5 m away from a space assigned for subjects activity. All three sensors were elevated roughly 1 m from the ground. In the large layout, the sensors were distributed at a similar shape and height, while 2.5 m away from the region of interest. Both layouts ensure that the subjects are visible in the field of view of the sensing system. The actual setup of the three sensors and subjects can be observed in Figure 2b.

#### 2.1.3. Environmental Temperature of Coventry-2018 Dataset

The pixels of LRIR array indicate temperature of area in front of the sensor. At the beginning of the data acquisition experiments, the ambient temperature of the surrounding room was measured to be 18° centigrade. However, during the time taken to perform the experiments and capture gestures, the ambient temperature of the surrounding room rose to 21°. The participants were wearing normal daily clothes in order to be compliant with a real-world scenario.

#### 2.1.4. Coventry-2018 Dataset Description

The dataset is composed of gestures that are distinguished into two categories in terms of the number of subjects. As shown in Table 1, the first category contains eight *single-subject* activities, while the second category contains seven *double-subject* activities. Single-subject activities refer to activities performed by one person. For example, a “Sit-Down” or “Stand Still” activity is performed only by one of the subjects in the field of view of the sensors. On the other hand, double-subject activities are performed by two people simultaneously. For example, in a “Walking Across” activity, two subjects walk in opposite directions in front of the sensors. In the experiments, three participants (with diverse body sizes and genders) were used. They act individually as a single-subject or in groups of two as double-subject. In the single-subject category, eight activities were performed by all three participants and each participant repeated each activity for 10 times. The eight single-subject activities are coded as AS1–8 in the small-layout scenario, and as AL1–8 in the large-layout scenario. In the double-subject category, seven activities (B1–B7) were performed by three participants pairs (2 out of 3 participants), and each participant pair repeated the activity 10 times. The double-subject activities were only performed in the large layout. Thus, there are a total of 480 single-subject records (240 in small layout and 240 in large layout), and 210 double-subject records (only in large layout).

### 2.2. Infra-ADL2018 Dataset

The *Infra-ADL2018* dataset also uses Grid-EYE sensors. This dataset was collected by researchers in Bristol Robotic Lab (BRL), University of West of England. It is used for verification of the feature representation and recognition methods derived from the *Coventry-2018* dataset. In addition, the *Infra-ADL2018* dataset was acquired in a different environment. This can provide useful insights regarding the applicability of the derived methods in this study.

**Figure 3:**
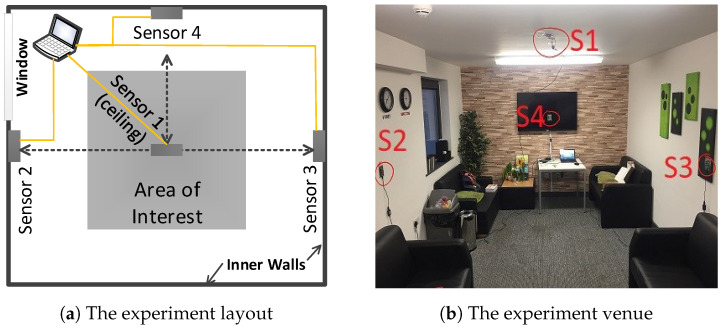
Illustration of the (**a**) experiment layout; (**b**) real experiment scenario, for the *Infra-ADL2018* dataset, where S1, S2, S3, and S4 refer to Sensor-1, Sensor-2, Sensor-3, and Sensor-4 respectively.

Compared with the *Coventry-2018* dataset, the *Infra-ADL2018* dataset presents four main differences: (1) One more sensor is deployed from the ceiling. (2) Besides the single-subject category (containing 9 activities) and double-subject category (containing 10 activities), the *Infra-ADL2018* contains an extra *three-subject* category. Similarly in this dataset, the single-subject and double-subject activities refer to those performed by one or two participants, respectively. In the case of three-subject activity, three participants performed the “Free Movement-Stand Still” activity in front of the sensors simultaneously, so that two subjects performed free movements and the third subject was standing still. This latter category contains only 2 activities, as shown in Table 2. (3) There were a total of nine participants. (4) Only one layout size was considered in the *Infra-ADL2018* dataset, as illustrated in Figure 3. Each activity was repeated three times by each participant or combination of participants. This led to 243 samples for single-subject activities, 240 samples for double-subject activities, and 18 samples for triple-subject activities.

## 3. Data Analysis

This section focuses on the analysis strategies employed for the LRIR datasets. Since the acting duration is not equal for different activities and participants, there is a diverse range of records duration in both of the *Coventry-2018* and *Infra-ADL2018* datasets. Thus, the records are equalized before being used for activity recognition for both analysis strategies based on (1) feature extraction from the 2D spatiotemporal maps and classification and (2) DNN applied on video streams of 8×8 LRIR frames based on convolutional LSTM.

**Figure 4:**
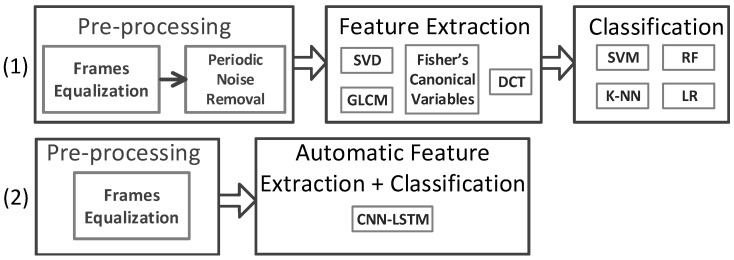
The data processing flow for the two analysis strategies.

As stated earlier in Section 1, the 2D spatiotemporal maps, used in the first group of analysis strategy, suffer from horizontal and vertical periodic noise, as shown in Figure 1b. Therefore, a periodic noise removal strategy is developed to alleviate the effect of noise in the developed 2D spatiotemporal arrays after frame equalization. This is performed to improve the activity recognition. This noise removal strategy was not required for the 8×8 frames used in the DNN strategy. Figure 4 shows two flowcharts depicting the procedures of the two analysis strategies considered in this paper.

### 3.1. Data Preprocessing

#### 3.1.1. Frames Equalization and Vectorization

The duration of the gestures captured by the sensors varies between 2 and 28 s. The average number of recorded frames for different activities and subjects is 69 and the standard deviation is 27 frames. After some primary tests, it was observed that equalizing the number of frames into 40 for all activities and subjects is the best compromise in terms of accuracy for HAR. Therefore, to implement the feature extraction and training processes, the records are equalized to 4 s (equivalent to 40 frames) for all activities in the *Coventry-2018* dataset. For the records longer than 4 s, extrapolation is used by removing frames at regular intervals. In the case of records shorter than 4 seconds, a new frame is added between two already existing frames. The newly added frame is computed by averaging those two frames. The same equalization strategy is applied on the *Infra-ADL2018* dataset.

Frame equalization yields 40 frames per activity with 8×8 temperature pixels. Considering the low resolution of IR images and low level of correlation between local pixels, the individual pixels can be considered as independent variables. Therefore, in order to form the 2D spatiotemporal maps shown in Figure 1b in Section 1, each 8×8 frame is vectorized. This is performed for all frames of each activity. Thus, the equalized 4-second records of each activity are reformed into a 40×64 2D spatiotemporal array. This 2D array can be treated as an image. In the analyses performed in this paper, the 2D spatiotemporal arrays are formed for both *Coventry-2018* and *Infra-ADL2018* datasets. This strategy will be compared later with time series analysis of the 8×8 frames, explained in Section 3.4, and its efficiency will be demonstrated in the results section.

**Figure 5:**
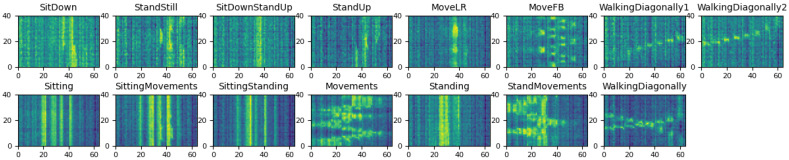
Illustration of the 2D spatiotemporal maps for the single-subject activities (**first row**) and double-subject activities (**second row**) of the *Coventry-2018* dataset.

**Figure 6:**
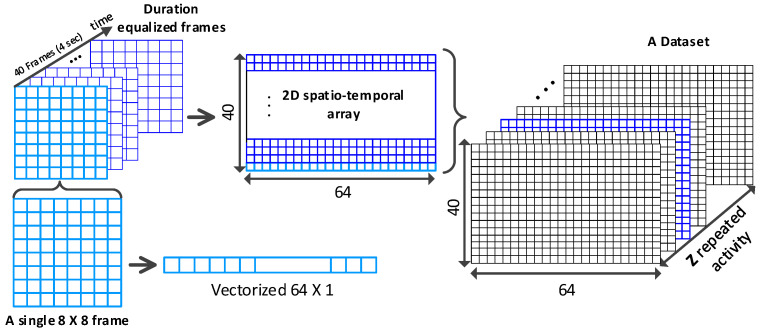
LRIR frames vectorization and concatenation (**left**) for shaping a 2D spatiotemporal array per activity (**middle**) and stacking all 2D maps for all activities in the dataset (**right**).

In the case of the *Coventry-2018* dataset, the equalized and vectorized data consist of a 3D array of size 240×40×64 for single-subject activities in small layout, a 3D array of size 240×40×64 for single-subject activities in large layout, and a 3D array of size 210×40×64 for double-subject activities in large layout. To simplify the writing and avoid confusion, the 3D array size is considered as Z×40×64. Z=240 is used for the single-subject data (in both small and large layout), and Z=210 for the double-subject data (only in large layout). An example of the equalized and vectorized LRIR frames is visualized in Figure 5 for eight classes of single-subject and seven classes of double-subject activities from *Coventry-2018* dataset. They are the 2D spatiotemporal maps used for activity recognition in this paper. The LRIR frames vectorization process is also shown in Figure 6.

#### 3.1.2. The Proposed Periodic Noise Removal Algorithm

In order to improve the activity classification accuracy, having high-quality data is important. The 40×64 2D spatiotemporal maps formed for each activity show periodic noise effect in both horizontal and vertical directions. This can be observed in Figure 1b. The periodic noise includes both horizontal and vertical stripes, though the vertical noise is visually more clear. The reason can be due to the low resolution of the frames that cannot encode small local variations. Then, the same value is assigned for adjacent pixels with a small level of variation. For example, the horizontal noise stripes are formed due to similarity of vectorized sequential frames. This can occur, for example, when an activity includes slow movements or still condition. On the other hand, the vertical noise stripes correspond to some unchanged pixels over all 40 frames, such as pixels that have never been occluded by the subjects. As can be seen, these effects are prominent in the arrays, and the classification tests demonstrated that they can negatively affect the classification accuracy in both training and validation stages.

In order to alleviate the effect of noise, 2D discrete Fourier transform (DFT) is used. It is a digitized version of the Fourier transform, such that it contains sampled frequencies of an image rather than all continuous frequency information. The 2D DFT of an image array is a 2D array of the same size. Each pixel in this 2D array corresponds to the coefficient in a specific frequency. They show the main spectral information of the image. The equation for 2D DFT is shown in (1).

(1) $$F\left[u,v\right]=\sum_{m=0}^{M-1}\sum_{n=0}^{N-1}X\left[m,n\right]e^{-j2\pi (u\frac{m}{M}+v\frac{n}{N})}$$

In (1), *M* and *N* show the number of rows and columns of *X*, and X[m,n] is one pixel in the original image, which is the 2D spatiotemporal map in this work. F(u,v) is the element in row *u* and column *v* of the output array *F*. This 2D DFT equation computes each element, F(u,v), in the spectral domain by multiplying the spatial image *X* with the corresponding sinusoidal and cosinusoidal base functions and summing up the result. For example, F(20,32) describes the information in zero frequency called DC. If *u* and *v* are set to zero in (1), the DC component is calculated as the average of the gray levels in the 2D spatiotemporal map. In this work, 2D DFT is calculated for each 40×64 2D spatiotemporal map, which is illustrated in Figure 6. Although F(u,v) is a complex value matrix, the amplitude of the complex coefficients are considered for further processing. Due to the large dynamic range of the DFT coefficients, the logarithm function is applied, yielding a 2D power spectrum array.

**Figure 7:**
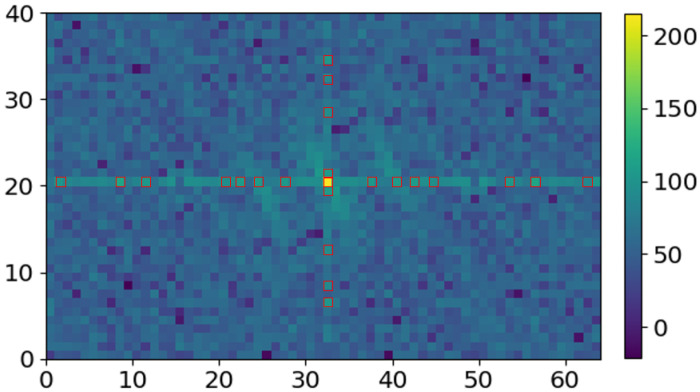
The 2D power spectrum with prominent symmetric peaks on central horizontal and vertical stripes highlighted with red color edges, corresponding to the vertical and horizontal periodic noise on the 2D spatiotemporal maps, respectively.

Next, the resulting power spectrum in the DFT domain is visualized, as shown in Figure 7. Due to the Hermetian symmetry property of DFT, the elements in F[m,n]=F(M−1−m,N−1−n), such that there is symmetry about $\left(\frac{M-1}{2},\frac{N-1}{2}\right)$, the central pixel in the 2D DFT array. For more information, the readers are referred to [20]. The coefficients in the central horizontal and vertical lines in this 2D spectrum are considered for periodic noise removal, as previously used in other works [21,22,23]. As expected, there exist pixels with noticeable symmetric peak values with respect to the center in both directions of the central horizontal and vertical stripes, as shown with red boundaries in Figure 7. These symmetric peaks indicate periodic effect of noise. The horizontal periodic noise corresponds to the peaks in vertical stripes coefficients and the vertical periodic noise is connected to the peaks in horizontal stripes, as shown in Figure 7.

In order to reduce the noise, the peaks should be identified and their values should be changed into a lower level over the central stripes. This can be achieved by thresholding the pixel values over the central stripes in the 2D power spectrum array so that pixels higher than a threshold will be numerically reduced. The DC component is not considered for thresholding as it represents zero frequency, or, in other words, shows the average values of the elements in the array and is not responsible for the noise. Two early issues regarding the choice of threshold values for changing the peak values should be addressed.

The first issue is the strategy for selection of an appropriate threshold value for each horizontal and vertical direction. Two possible methods can be considered in this case: ***(1)*** A unique constant threshold value for each direction based on the overall mean of the horizontal and vertical stripes over all 2D power spectrum arrays for all activities; and ***(2)*** a variable calculated based on the individual power spectrum arrays’ statistics for the threshold value.

The first method was tested statistically. For this aim, the mean values over the horizontal and vertical stripes (excluding the DC component) $\mu_{h}$ and $\mu_{v}$ for all activities of the three sensors (of *Coventry-2018* dataset) were calculated. Under the null hypothesis that all power spectral arrays have similar mean values (over the stripes) for each of the horizontal and vertical directions, a one-way ANOVA test was performed. The p-value rejects the null hypothesis, and, thus, general thresholds (for the two directions) based on the stripes’ mean values cannot be used for all 2D arrays. Therefore, the second method is considered and the threshold values are customized for each 2D power spectrum array over the two directions. For this aim, a supervised strategy will be introduced later in this section.

The second initial issue is regarding finding an appropriate strategy for reduction of the numerical values of the identified peaks based on thresholding. Two possible options for replacing the values of the identified peaks are (1) replacing with 0, and (2) replacing by a statistical measure computed based on the mean of the elements at each horizontal or vertical stripe, $\mu_{h}$ and $\mu_{v}$. By performing experiments, we found that the latter strategy resulted in better activity recognition accuracy. Therefore, for replacing the identified peaks values, the statistical approach based on the mean and standard deviation will be considered.

After addressing these two initial issues, as mentioned earlier, a supervised algorithm is developed for finding the best parameters for noise removal. This includes the thresholds and the appropriate number of pairs of identified peaks whosevalues should be reduced. For this aim, the threshold parameters $T_{h}$ and $T_{v}$ for each horizontal and vertical direction are formulated based on the mean and the standard deviation of all the coefficients in the corresponding stripes, as follows:

(2) $$T_{h}=\mu_{h}+i_{1}*0.5*\sigma_{h};T_{v}=\mu_{v}+i_{1}*0.5*\sigma_{v}$$

where $i_{1}$ is an integer value within the range {−2, 8} that is learned based on a supervised strategy. In addition, as there exists a number of pixel pairs greater than the threshold, it needs to be decided how many of them should be reduced to achieve the highest accuracy. The number of pairs of pixels to be changed depends also on the threshold value. In other words, the smaller the threshold, the bigger the number of pixel pairs violating the threshold. Overall, the parameters $T_{h}$ and $T_{v}$, and number of pairs of pixels, $Num_{h}$ and $Num_{v}$, that influence the quality of noise removal, are found based on a supervised learning strategy in order to achieve optimum accuracy.

The supervised learning strategy is based on the K-fold cross-validation (CV) model selection technique, so that 10-fold CV is used to find the optimum parameters $T_{h}^{*}$/$T_{v}^{*}$ and $Num_{h}^{*}$/$Num_{v}^{*}$ for each of the horizontal and vertical directions. For this aim, candidate sets of values are formed for these parameters, $T_{CandSet}$ and $Num_{CandSet}$, first. The $T_{CandSet}$ is formed based on the variable $i_{1}$, as shown in (2), and the $Num_{CandSet}$ is formed based on the maximum pairs of pixels in each direction. Thus, based on the CV loop, a 3D array of validation accuracy of size $K\times length\left(T_{CandSet}\right)\times length\left(Num_{CandSet}\right)$ is calculated.

In each CV iteration, one candidate threshold value from $T_{CandSet}$ is used to find the pairs of peaks that violate the threshold (in the corresponding direction). Then, one candidate number of pixel pairs is selected from $\left(Num_{CandSet}\right)$ and is used to select a number of highest peaks. The selected peaks values are reduced to $\mu_{h}$ or $\mu_{v}$ depending on the direction. Next, based on the 2D inverse DFT (iDFT) function shown in (3), the denoised 2D spatiotemporal maps are calculated.

(3) $$X\left(m,n\right)=\frac{1}{MN}\sum_{u=0}^{M-1}\sum_{v=0}^{N-1}F\left[u,v\right]e^{j2\pi (u\frac{m}{M}+v\frac{n}{N})}$$

The image with alleviated horizontal noise is merged with the image with reduced vertical noise, by averaging the two maps calculated based on the inverse transform. After repeating this for all training and validation images, they are used to perform activity recognition in the next step.

**Figure 8:**
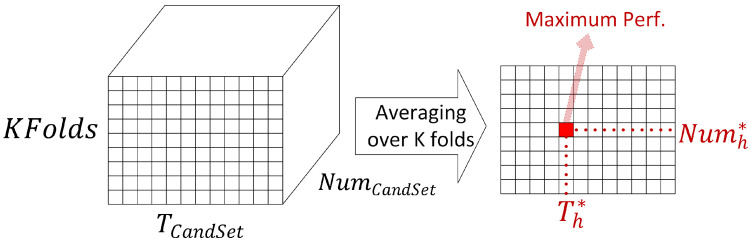
Illustration of the 3D array of validation accuracy for the horizontal stripe parameters and the 2D array of average validation performance obtained by averaging over K-folds of the 3D tensor for selection of $T_{h}^{*}$ and $Num_{h}^{*}$ parameters.

Then, the recognition performance for both train and validation sets is calculated. This process is iterated inside the three nested loops for all *feasible combinations* of the three parameters, namely, K (of CV loop), $T_{CandSet}$, and $Num_{CandSet}$ (of the candidate sets). This is repeated for both horizontal and vertical stripes parameters. Based on these three nested loops, the reason for the aforementioned dimension of the performance arrays to be three for both training and validation sets can be explained. The three-dimensional array is also referred to as training and validation tensor. Figure 8 shows the 3D tensor of validation accuracy for the horizontal stripe parameters as well as the averaged 2D array of validation performances over the K-folds. In the validation and training tensors, only the elements corresponding to the *feasible combinations* of candidate thresholds and number of pixel pairs are filled. This is explained further in the following.

Not all combinations of the candidate thresholds and number of pairs of pixels are feasible for noise removal, because the number of violating peaks of pixel pairs changes based on the threshold value such that the smaller threshold increases them and the bigger threshold value reduces their number. Then, there might not exist any candidate pair of peaks for some combinations of the $T_{CandSet}$ and $Num_{CandSet}$. As a result, the elements of the validation tensor corresponding to those *infeasible combinations* of the candidate thresholds and the number of pixel pairs are empty and will be ignored.

**Figure 9:**
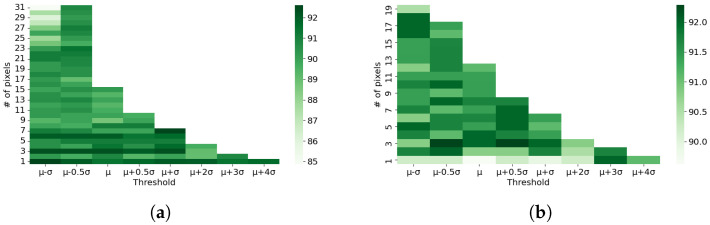
Heat maps of average CV validation accuracy array for selection of optimum *T* and Num parameters for (**a**) horizontal and (**b**) vertical stripes.

As shown in Figure 8, by averaging the 3D tensor of validation performances over the K-folds, a 2D array of validation accuracy is obtained. The size of this 2D array is $length\left(T_{CandSet}\right)\times length\left(Num_{CandSet}\right)$ for each of the horizontal and vertical stripes. The 2D array shows the variation in performance for different feasible combinations of the candidate parameters $T_{CandSet}$ and $Num_{CandSet}$, shown as two heat maps in Figure 9 for the two directions. Then, the highest performance in the 2D arrays corresponds to the optimum parameters $T_{h}^{*}$/$T_{v}^{*}$ and $Num_{h}^{*}$/$Num_{v}^{*}$. Using these parameters, the vertical and horizontal noises are removed, resulting in two denoised images after applying 2D iDFT transform. The two denoised images are merged by averaging. This is performed for all training images in the dataset, making them ready for activity recognition in the next step.

**Figure 10:**
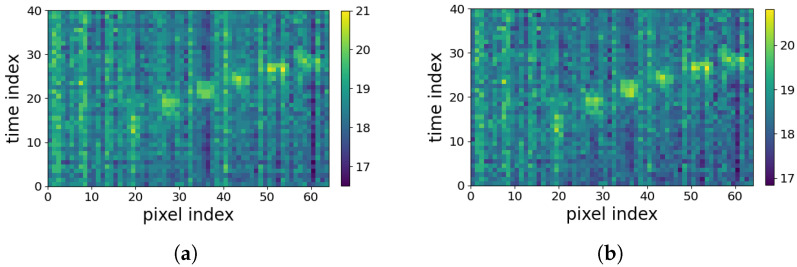
Illustration of (**a**) an original 2D spatiotemporal map with horizontal and vertical periodic noise; (**b**) noise-reduced 2D map using the proposed algorithm.

Algorithm 1 shows the step-by-step procedure using training data. Thus, the required parameters, $T_{h}^{*}$/$T_{v}^{*}$ and $Num_{h}^{*}$/$Num_{v}^{*}$, are derived and applied to the test images. A sample noisy image and its corresponding denoised image using this algorithm are illustrated in Figure 10.

**Algorithm** **1:** **DFT-based periodic noise removal.** **Input:** Training data ($X_{tr},Y_{tr}$), Number of folds *K*. 1. Divide data into K training and validation sets: $xtr_{k}$, $ytr_{k}$, $xv_{k}$, $yv_{k}$. 2. Repeat the following steps for both horizontal and vertical stripes represented as horizontal/vertical for showing all parameters.
 3. Form the $T_{CandSet}=2,\ldots ,8$ and $Num_{CandSet}=$1,…, Maximum number of pixel pairs.
 4. (1) **For** k = 1, …, K
   (2) **For** $i_{1}=1,\ldots ,length\left(T_{CandSet}\right)$
    (3) **For** $i_{2}=1,...,length\left(Num_{CandSet}\right)$
     (4) **For** j = 1, …, number of images in $xtr_{k}$ and $xv_{k}$
      -Compute the power spectrum coefficients of the $j^{th}$ image,
      -Compute $\mu_{hj}$/$\mu_{vj}$, $\sigma_{hj}$/$\sigma_{vj}$
      -Find pixel pairs in horizontal/vertical stripes
      $>T_{hj_{i_{1}}}=\mu_{hj}+T_{CandSet}\left(i_{1}\right)*0.5*\sigma_{hj}$/
      $T_{vj_{i_{1}}}=\mu_{vj}+T_{CandSet}\left(i_{1}\right)*0.5*\sigma_{vj}$,
      -Reduce the first $i_{2}^{th}$ greatest pixel pair values to $\mu_{hj}$/$\mu_{vj}$,
      -Compute the denoised image using 2D iDFT,
     **End Loop 4**
     -Use the denoised image sets $xtr_{dk}$ and $xv_{dk}$
     to classify the activities and calculate the
     element ($k,i_{1},i_{2}$) in the training and validation
     performance arrays, $Prf_{tr}$ and $Prf_{v}$.
 **End Loop 3,2,1**
 5. Average the $Prf_{tr}$ and $Prf_{v}$ arrays over the K-folds
 6. Find the highest validation accuracy corresponding to
 the optimum threshold $T_{h}^{*}$/$T_{v}^{*}$ and number of pixel pairs $Num_{h}^{*}$/$Num_{v}^{*}$.
 **Output**: $T_{h}^{*}$/$T_{v}^{*}$ and $Num_{h}^{*}$/$Num_{v}^{*}$


### 3.2. Feature Extraction Methods

As stated in Section 1, in this paper, two main categories of feature extraction approaches are considered and applied on the denoised 2D spatiotemporal maps dataset. These include (1) orthogonal transformations based on singular-value decomposition (SVD) and the Fisher’s canonical variables, and (2) texture analysis in frequency domain based on 2D DCT and in spatial domain based on GLCM.

SVD is one of the most common methods for transforming data into a lower-dimensional space based on calculating and sorting the directions of highest variance [24,25]. It is used to transform the vectorized 2D maps of 40×64=2560 features to a lower dimensional space. This is achieved by computing the transformed features into a lower dimension orthogonal feature space $Z_{SV\;D}=S_{Z\times 2560}V_{1:k}$, where $S_{Z\times 2560}$ is the original matrix of samples formed based on the vectorized 2D map features, Z denotes the number of samples, and $V_{1:k}$ are the first *k* eigenvectors corresponding to the highest singular values. In this work, k=45 eigenvalues are used, describing more than 95% of the variance of the data. This means that the feature dimension is reduced from 2560 to 45. A linear combination of all variables is used to transform data to a much smaller orthogonal feature space.

Fisher’s canonical variables are a widely-used supervised method for dimensionality reduction [26,27,28]. The method transforms the vectorized features (2560 in our work) to a maximum of Q−1 eigenvectors, where *Q* refers to the total number of classes. The overall aim of the method is to maximize the Euclidean distances between samples of differing classes, and minimize the distances between samples belonging to the same class. This is achieved by solving a generalized eigendecomposition problem using the between-class covariance matrix using all data as well as sum of each class covariance matrix (within-class covariance). The found eigenvectors are used to transform samples to a new orthogonal space of dimension Q−1. Considering the *Coventry-2018* dataset, a 15-class scenario is used as a combination of single-subject (8 classes) and double-subject (7 classes) activities. This yields a maximum of 14 eigenvectors extracted by the Fisher’s method.

The 2D DCT can be considered as a texture analysis strategy that transforms 2D inputs (signals or images) to the frequency domain [29]. The smooth variations correspond to low-frequency information in spectral domain while sharp variations correspond to the high-frequency coefficients. The method sorts the frequencies in ascending order, which allows filtering high- or low-frequency coefficients. This allows a limited number of features to be extracted. In our work, each 2D 40×64 feature map is divided into 40 local patches of size 8×8, and 2D DCT is applied to these local patches. Then, six coefficients are extracted in a zigzag pattern from the top-left corner of the 2D DCT array corresponding to each local patch. This results in a data matrix of Z×40×6=Z×240, with 240 features and Z number of samples.

GLCM is another image texture analysis technique [30]. It aims to quantify the frequency of occurrence of different combination of gray values in an image, which shapes image patterns.For example, determining how often a pixel with a given intensity $L_{a}$ occurs at a distance *d* and angle Θ to another pixel with intensity $L_{b}$. This will be recorded in a matrix and some statistics are then derived from the matrix. Considering the nature of GLCM, it is applied to the 40×64 2D maps. Four second-order statistics are then calculated from the GLCM matrix, including correlation, contrast, dissimilarity, and energy. For more information, refer to [30]. In this work, first, the 40×64 images are divided into five local patches of size 8×64, and then, for each local patch, GLCM matrices are calculated for two different distances (one and three number of pixels) and three different angles, 0°, 45° and 90°. This results in 5×2×3 GLCM matrices. For each GLCM matrix, four GLCM statistics are calculated. Then, for each 40×64 map, 120 texture features are extracted based on GLCM analysis, resulting in GLCM features of size Z×120 for the 3D array datasets Z×40×64. Z denotes the number of samples.

### 3.3. Classification Methods for Activity Detection

In this work, four traditional classification methods are used: SVM, RF, k-NN, and LR. All four techniques are supervised and show suitability for multiclass classification problems.

### 3.4. Deep Neural Networks (DNNs) for Activity Recognition

DNNs are powerful models capable of learning a hierarchy of features from low-level ones to automatically build high-level ones. Thus, the feature extraction is automated, which eliminates the need for handcrafted feature extraction. In our work, the LRIR data can be treated as a sequence of 8×8 IR images, which leads to the exploration of CNN and LSTM models. Therefore, the CNN layers are used for extracting features, while the LSTM layers classify the extracted outputs [31,32,33].

The robustness of the model is evaluated to discover the most optimum combination of CNN and LSTM layers. As a result, three convolutional and two LSTM layers are considered for the CNN-LSTM architecture. The inputs are samples that each consist of 40 frames of size 8×8 and their corresponding activity label.

**Figure 11:**
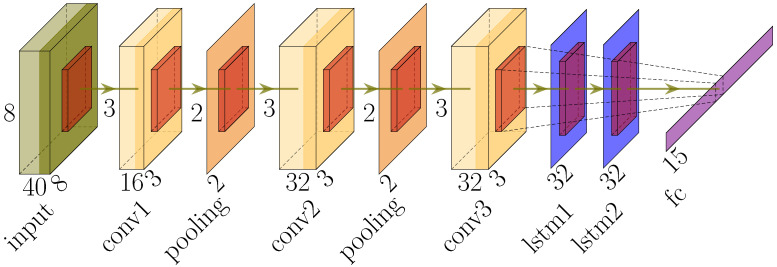
Illustration of the CNN-LSTM architecture.

A total of 1000 number of epochs are used for training the model, and the batch size of 32 achieved better results compared to other values, 64 and 128. The developed CNN-LSTM architecture with the remaining hyperparameters is represented in Figure 11. The first convolutional layer has 16 filters of size 3×3. Regarding the second and third convolutional layers, 32 filters with the same size are used. The two LSTM layers both have 32 filters.

## 4. Evaluation and Results

The datasets in this study are divided into training (75%) and testing (25%). The testing accuracy is used to evaluate and compare the models. In this section, the following experiments are presented:

- Noise-reduction test—the test is performed on the *Coventry-2018* dataset to compare the models’ performances on noisy data and noise-reduced data.
- Comprehensive model comparison test—all combinations of feature extraction and classification methods, as well as CNN-LSTM, are compared based on the *Coventry-2018* dataset to discover the most optimum modeling strategy. Then, the best model is applied to different scenarios of the *Infra-ADL2018* dataset.
- Layout-sensitivity test—the test is performed on the *Coventry-2018* dataset to compare the effect of sensor distance to the subject using the small-layout and large-layout data.
- Model-generality in terms of layout—the test is performed to evaluate the generalization of a model trained on one layout datum (large or small), when tested on another layout datum. In addition, a mixture of small-layout and large-layout samples is applied as the second scenario evaluation. The *Coventry-2018* dataset is used for this test.
- Subject-sensitivity test—the test is performed to evaluate the effect of number of subjects (one or more) on the performance of the models. The *Coventry-2018* dataset and *Infra-ADL2018* dataset are used for this experiment.
- Optimum sensor test—the test is performed to identify the optimum number and position of sensor(s) that can give the highest performance. All individual sensors and combinations of them are used for this experiment using the *Coventry-2018* and *Infra-ADL2018* datasets.

### 4.1. Result of Periodic Noise Removal

In order to evaluate the effect of the proposed periodic noise removal strategy, the classification results of data before preprocessing and after preprocessing are compared. For this aim, the *Coventry-2018* dataset for a 15-class problem including both single-subject and double-subject activities of Sensor-1 was used. Sensor-1 was selected since it was found to be the optimum sensor based on the test results that will be presented later in this section.

**Figure 12:**
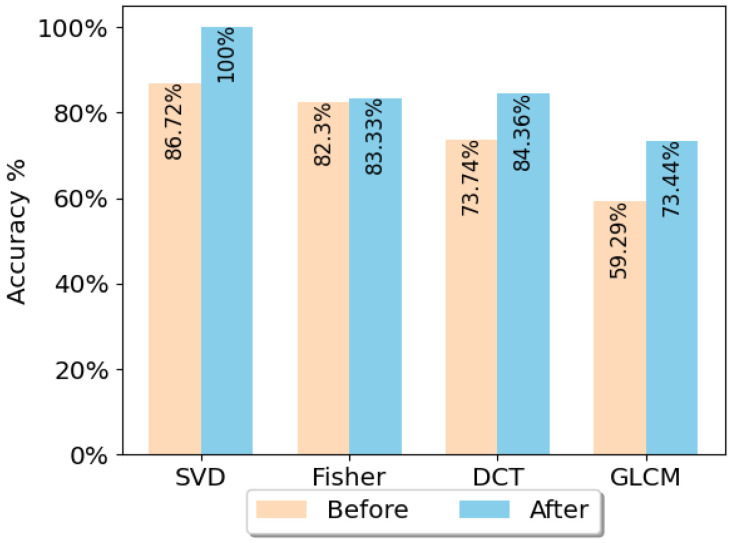
Comparison of the classification performances before denoising and after applying the DFT-based periodic noise removal algorithm, using different feature extraction methods and LR classification. The *Coventry-2018* large-layout data (15-class problem) of Sensor-1 was used.

The four feature extraction methods, SVD, Fisher’s method, DCT, and GLCM, with LR were used to train the classification models. The LR classifier was chosen because it was one of the best classifiers compared to others in terms of accuracy. This will be shown in the next section. The models were trained using the raw data (before denoising) first. Then, the same modeling strategies were applied on the denoised datasets. The results of this experiment show an increase in accuracy. Figure 12 shows the result of classification. The *Coventry-2018* large-layout Sensor-1 data was used for these tests. The results show improvement in the classification performance in all cases.

### 4.2. Comprehensive Comparison of Activity Recognition Techniques

This section compares the capability of different combinations of feature extraction and classification methods described in Section 3.2 as well as the CNN-LSTM in activity recognition. The idea is to evaluate the methods using the most challenging dataset at hand. Therefore, instead of evaluating based on the 8-class data (of single-subject activities) or 7-class data (using double-subject activities), the 15-class dataset, including both single-subject and double-subject activities from the *Coventry-2018* large-layout Sensor-1, are considered in this section. The handcrafted feature extraction methods are SVD, Fisher’s canonical variables, DCT, and GLCM. The investigated classifiers include SVM, RF, k-NN, and LR.

K-fold CV (K=10 in this work) was used in order to find the optimum parameters for the classifiers. The main parameters found based on K-fold CV for the models include a linear kernel for SVM, 100 trees for RF, k=1 for k-NN, and the limited-memory Broyden–Fletcher–Goldfarb–Shanno (BFGS) optimization solver for LR. The dataset was divided randomly into training and test set 10 times to perform the tests, and the average and standard deviation of the results are reported.

Table 3 presents the results achieved based on different feature extraction–classifier combinations as well as the CNN-LSTM model. The results show that the applied methodologies were successful in discriminating activities. The highest accuracies are based on the unsupervised SVD feature extraction and CNN-LSTM, where the former achieved 100% when using LR classifier. Although the CNN-LSTM model is more complicated compared to SVD+LR in terms of computation time, its performance is not higher.

Based on the classification results for the *Coventry-2018* dataset, SVD feature extraction with LR classifier was selected as the optimum modeling strategy. Therefore, it is used for the rest of the experiments in this paper. Then, it is also applied on the *Infra-ADL2018* dataset. In this dataset, among the three categories of single-subject, double-subject, and three-subject activities, the number of samples for the latter are very limited (18 samples per sensor). This makes the model training very difficult. Therefore, in this paper, only the single-subject (9 classes) and double-subject (10 classes) activities data were used separately, as shown in Table 4. In addition, all three groups, including single-, double-, and three-subject activities, of data were used to develop a model for classification of all activities (21 classes) using each sensor’s data. The achieved results show the robustness of SVD and LR on this dataset.

### 4.3. Layout-Sensitivity Test Results

**Figure 13:**
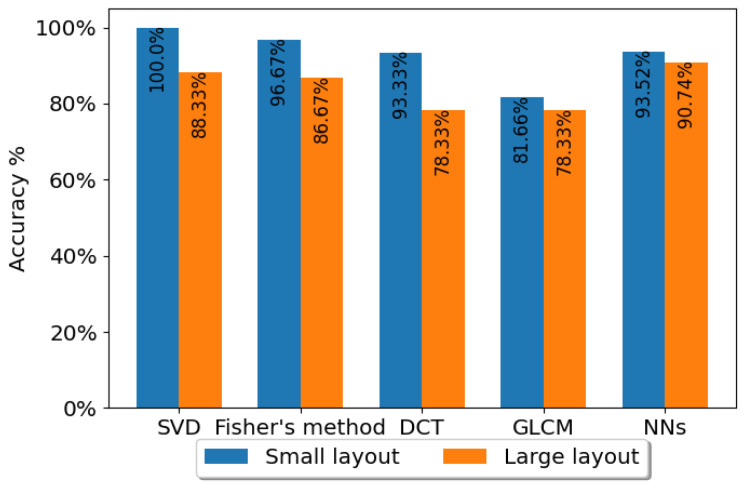
Comparison of the effect of small-layout and large-layout data in classification performance. The LR classifier with the four feature extraction methods are applied on the single-subject activities for small-layout and the single-subject activities for large-layout, as well as the CNN-LSTM using *Coventry-2018* Sensor-1 large- and small-layout datasets.

One of the concerns regarding the use of LRIR sensors is sensitivity in their distance to the target. In this section, the impact of distance when using the LRIR sensor for human target activity recognition is presented based on the experiments. For this aim, the single-subject small layout (1.5 m away from area of interest), including 240×40×64 data, and the single-subject large layout (2.5 m away from area of interest), also including 240×40×64 samples, were utilized. The performance of the four SVD, Fisher, DCT, and GLCM feature extraction methods using the LR classifier as well as the CNN-LSTM model were compared. In Figure 13, the recognition performances are shown.

As expected, the accuracy for the small layout is higher as the subject body profile covers more pixels. Nevertheless, the difference in the accuracy is not significant, which showcases that gestures are correctly classified in both cases. This indicates that the applied techniques, including preprocessing, feature extraction, and classification, are applicable for sensor settings placed at different distances with respect to the subject.

### 4.4. Model-Generality in Terms of Layouts

A classical machine learning problem is determining whether a model can generalize for different scenarios. In the *Coventry-2018* experiments, two facts are worth noticing: ***(1)*** there are small and large layouts; ***(2)*** the room temperature increased during the experiments when collecting the large-layout data. These two facts can be utilized to test the models’ generalization when having unseen layout condition after training. For this aim, two different scenarios are arranged.

1. In the first experiment of the first scenario, the model which was trained using the single-subject Sensor-1 data in small layout was tested on the single-subject Sensor-1 data in large layout. The second experiment was also repeated for the large layout as train and small layout as test. The distances from sensors to the subjects are different. In addition, the average and standard deviation of the pixels for the small layout are 17.47±1.21, while for the large layout, they are 18.65±0.72. All combinations of feature extraction and classification methods as well as CNN-LSTM were tested. GLCM for feature extraction with LR for classification achieved the best accuracy in both experiments using Sensor-1. In the first experiment, the accuracy was 71%. In the opposite scenario, where the large layout is used for training and the model is evaluated on the small layout, the testing accuracy was 64.58% using the same feature extraction and classification strategy. This shows that the (GLCM + LR) can still generalize and show intermediate levels of results of 71% for small-layout training and large-layout testing. This is a positive indication that such systems still work in the case of changes in the original settings such as displacement, etc.
2. In the second scenario, the single-subject data from both small layout (240 samples) and large layout (240 samples) were mixed, which represents the moderate scenario. Similar to the previous experiment, all classification models were tested in the pursuit of the most optimum model. As a result, the most accurate model was the automatic feature extraction and classification with the deep CNN-LSTM architecture, achieving 94.99%±1.66 average testing accuracy. Considering the very high accuracy, this experiment shows that having approximately equal distribution of data from both layouts in the training phase provides a very rich representation of the data, leading to a strong generalized model.

### 4.5. Sensitivity to the Number of Subjects

In this section, the sensitivity of the algorithms in detection of the activities with different number of subjects is evaluated. For this aim, the single-subject dataset of size 240×40×64 and double-subject of size 210×40×64, both from the *Coventry-2018* large-layout settings of Sensor-1, were used for comparison. In addition, single-subject activities of size 243×40×64 and double-subject activities of size 240×40×64 from the *Infra-ADL2018* were also compared. Table 5 describes the test accuracy of LR classifier when applied on SVD, Fisher’s method, DCT, and GLCM features, as well as the CNN-LSTM structure. The graph shows that the double-subject activities outperform the single-subject activities for all five models developed from the two datasets.

### 4.6. Optimum Room Setup and Sensor Selection

**Figure 14:**
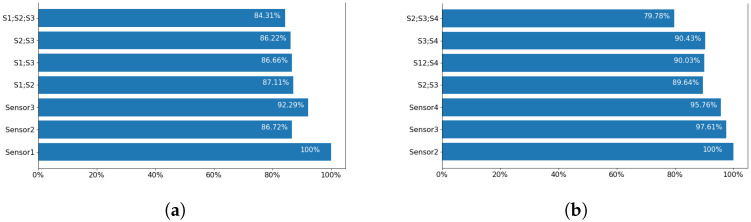
Classification accuracy for individual sensors and combinations of them for (**a**) *Coventry-2018* dataset and (**b**) *Infra-ADL2018* dataset.

The room setup was part of a comprehensive discussion prior to any experiment conduction. For *Coventry-2018* experiments, instead of using one sensor attached to the ceiling of the room, it was decided to use three sensors in total. Then, it appeared that the most successful results were achieved using Sensor-1 with a side view to the subjects. In order to identify the optimum sensor arrangement, the classification accuracies for different combinations of sensors were compared. The results of this comparison are shown in Figure 14 for both *Coventry-2018* and *Infra-ADL2018* datasets. In the case of the former dataset, as shown in Figure 2a, Sensor-1 and Sensor-3 are the side sensors and Sensor-2 is the front sensor in the scene. In the case of the *Infra-ADL2018* dataset, as shown in Figure 3a, Sensor-2 and Sensor-3 are the side sensors (comparable with Sensor-1 and Sensor-3 of the *Coventry-2018*) and Sensor-4 is the front sensor (comparable with Sensor-2 of the *Coventry-2018*). Then, these three sensors were used in the optimization experiments for comparison with the *Coventry-2018* dataset. In the case of the extra ceiling-mounted Sensor-1 of the *Infra-ADL2018* dataset, it was excluded from the sensor optimization experiments due to two reasons: (1) it did not achieve better results than the other sensors (2, 3, and 4), as shown in Table 4, perhaps because of the weaker silhouette view of the subjects from the ceiling; (2) there is no comparable sensor in the *Coventry-2018* dataset for that sensor.

For these experiments, the models were trained using the SVD feature extraction and LR classifier. The large layout of the *Coventry-2018* dataset was used, including both single- and double-subject activities. In the case of *Infra-ADL2018*, all 21 activities were used for evaluation.

As observed in these results, in both cases, the individual side sensors achieved highest accuracies. Sensor-1 from the *Coventry-2018* and Sensor-2 from the *Infra-ADL2018* show 100% prediction results. Similarly, the other two side sensors, including Sensor-3 of the *Coventry-2018* dataset and Sensor-2 from the *Infra-ADL2018* dataset, achieved the next highest accuracies. These show an agreement between the two datasets regarding the optimal use of side sensors for the dominant viewpoints, as it is promising in terms of accuracy and simplicity in the system design and computations for activity recognition. Although different combinations of the three sensors caused a decrease in the testing accuracy, they are still at a high level. This drop of the results might be also connected to the increase in the number of variables by including more sensor frames while having the same number of samples. This should be further explored in future studies by providing more samples for the experiments.

## 5. Discussion

### 5.1. Optimum Sensor Selection

By using multiple sensors, a systematic review of the performance of each sensor can be performed. As seen in Section 4, using Sensor-1 data, the best activity recognition accuracy was achieved. Based on this result, a conclusion might be derived on exclusion of the other two sensors, which simplifies both hardware and software requirements. However, due to the variations in the orientation of subjects with respect to a sensor in real scenario, the expected accuracy might vary depending on the subject’s direction with respect to the sensor, as seen for the three individual sensors’ results in Figure 14. Furthermore, as mentioned earlier, the temperature of the room rose from 18° centigrade to 21° over the experiments in the large layout. However, the classification models still remain accurate despite the higher temperature in the room.

**Figure 15:**
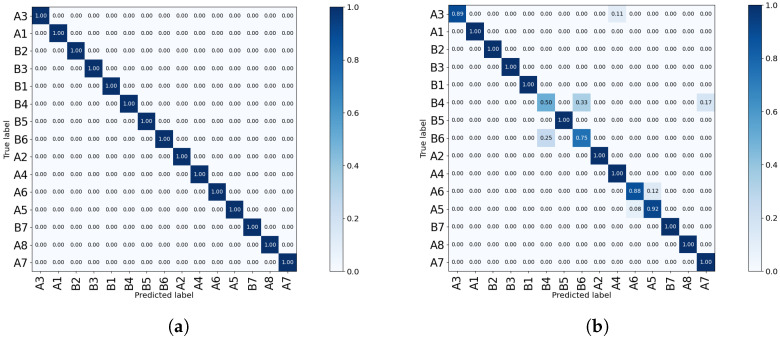
Comparison between the confusion matrices of the two side sensors, Sensor-1 (**a**) and Sensor-3 (**b**) of the *Coventry-2018* dataset. The large layout with single- and double-subject data was used for training a model based on SVD and LR.

**Figure 16:**
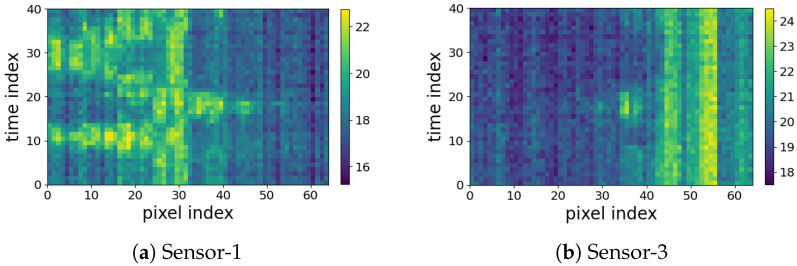
Comparison between *Coventry-2018* Sensor-1 (**a**) and Sensor-3 (**b**) for “Standing + Small Movements” gesture.

Another issue to investigate is the reason for the difference in the performance of the two side sensors, Sensor-1 and Sensor-3. While they are symmetric with respect to the subject’s position, Sensor-3 shows lower accuracy compared to Sensor-1. The confusion matrices shown in Figure 15 outline that the lower accuracy of Sensor-3 is due to the double-subject gestures such as B4 (Small Movements) and B6 (Standing; Moving). This is due to the fact that these gestures are not symmetric, as visualized in Figure 16. Furthermore, the confusion matrices in Figure 15 prove that the pair-subject gestures are problematic and this leads to lower performance of the data captured by Sensor-3.

### 5.2. Analysis of Model Accuracy

As stated in Section 1, one of the initial targets in this paper was a comprehensive comparison of supervised methods, including the group of feature extraction and classification as well as DNN based on CNN-LSTM, in the pursuit of discovering the most accurate model for LRIR data and activity prediction. Furthermore, having a diverse group of analysis methods helped in achieving reasonable accuracy in scene generalization experiments where both large-layout and small-layout data were used together. The obtained results demonstrated that specifically SVD was the most successful feature extraction method in activity detection using LR, RF, and SVM classifiers. This means that the most discriminating features lie along the directions with highest variance in orthogonal subspace. SVD uses the whole training samples of all classes to find the eigenvectors, that is, 75% of the total 450 number of samples (of the Coventry 15-class problem), 0.75×450=337. However, the Fisher technique requires computing the within-class covariances. Since there are only 30 samples in each of the 15 classes, then 0.75×30=22 training samples are used for computation of the within-class covariance matrices. This is very low compared to the large number of variables, 2560. This probably places SVD in a better rank condition when referring to the achieved accuracies. Another successful architecture was the CNN-LSTM model, where features were extracted from the CNN layers. Therefore, the LSTM layers learned the temporal variations of the sequences.

On the other hand, GLCM showed some level of success for model generalization when a model trained on small-layout data was used to classify samples of large layout. This is due to the nature of the GLCM in quantifying image texture patterns. With regard to SVD, the main directions of variations found in orthogonal subspaces might not match between the small and large layout. In addition, due to the changes in spectral variations of 2D arrays in the two layouts, a previously trained model on the compressed DCT coefficients of small layout does not work on large-layout data. However, a model trained on the texture patterns captured by GLCM for small layout identifies the same patterns in large-layout data and shows generalization capability. In addition to this, the deep CNN-LSTM architecture showed superiority when recognizing activities in the mixed model generalization scenario.

### 5.3. Data Augmentation

The number of existing samples per activity is 30 for the *Coventry-2018* and 27 for the single-subject activities of the *Infra-ADL2018* dataset. These are limited number of samples. Then, evaluating the models using a higher number of training samples can better reveal their capability and accuracy. To the best of our knowledge, there is no available dataset with similar level of diversity in the number of subjects and activities to the two datasets used in this paper. Therefore, to evaluate the optimum model using a larger number of training and test samples, data augmentation is considered. There are different strategies to artificially expand the size of samples. One group of methods are based on applying functions such as rotation, flipping, and changing image heights and width. However, this cannot be adopted for augmenting the 2D maps, and care must be taken in their augmentation to preserve the validity of the generated 2D spatiotempral maps. This is because they are shaped by vectorization and concatenation of the IR frames. The abovementioned augmentation methods can result in generation of frames including nonrealistic activities or silhouettes.

**Figure 17:**
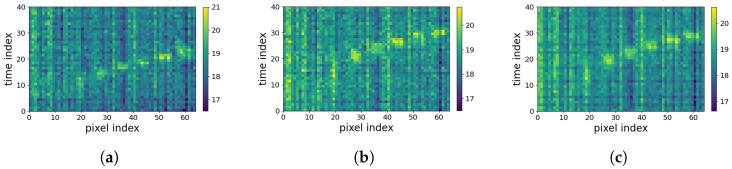
Illustration of the 2D spatiotemporal maps of subject 1 performing activity “Walking-Diagonally 1”: (**a**) repeat 1; (**b**) repeat 2; (**c**) the artificially generated 2D map using the (**a**,**b**) maps.

Therefore, in this paper, image augmentation was performed by interpolation of the 2D spatiotemporal maps. This was performed for the 15-class problem of the large layout, side Sensor-1 of the *Coventry-2018* dataset. For this aim, the average of two spatiotemporal maps of the same subject was calculated to generate a new spatiotemporal map. In total, 10 new maps were generated out of the 10 original maps of each subject for each activity. The order of the maps was selected as $\left(map_{1},map_{2}\right),\left(map_{2},map_{3}\right),\ldots ,\left(map_{9},map_{10}\right),$$\left(map_{2},map_{4}\right)$. This doubled the number of samples from 450 to 900. Figure 17 shows one example of the 2D maps from two repeats of a subject and an artificially generated map using the two repeats.

**Figure 18:**
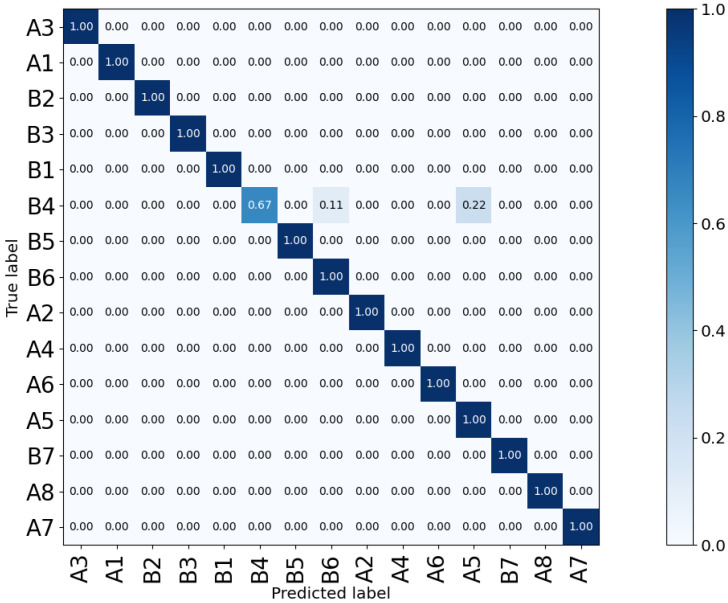
Confusion matrix describing the prediction accuracy based on the augmented dataset *Coventry-2018* for the 15-class problem using the large-layout Sensor-1 data.

Next, the augmented data are randomly divided into train (75%) and test (25%). This is followed by periodic noise removal based on the proposed FFT-based algorithm. To estimate the classification accuracy, the most optimum model, SVD+LR, is used. The resulting confusion matrix for the test data is shown in Figure 18. While the training accuracy remained at 100%, compared to the original samples’ test results shown in Figure 15a, there is a slight reduction in the new overall test accuracy (98.66%). This is due to the increased diversity in the test samples. Interestingly, the few wrongly classified activities belong to the same challenging class of B4 (Small Movements) activity, which was also wrongly classified for some samples of Sensor-3 previously (shown in Figure 15b). The proposed periodic noise reduction method increased the overall testing accuracy from 96% to 98.66%. The results achieved after data augmentation confirm the accuracy and reliability of the optimum methods found for HAR using LRIR data in this paper.

## 6. Conclusions

In this paper, the problem of human activity recognition using low-resolution IR sensors was addressed. Experiments were conducted in large-layout and small-layout settings by changing the distance from sensors to the subjects. For the first time, our generated dataset, called *Coventry-2018*, was used for HAR. In addition, a previously created dataset, *Infra-ADL2018*, was analyzed for comparison. Furthermore, the experiments included single-subject activities as well as double-subject activities. A novel supervised denoising algorithm based on 2D DFT was proposed for removing the horizontal and vertical periodic noise from the 2D spatiotemporal image maps formed by concatenation of the vectorized frames over the activity period. The use of the proposed strategy improved the activity classification accuracy. For classification of the activities, two main approaches were used: the feature extraction for dimensionality reduction followed by classification modeling and a deep learning strategy. The former strategy included SVD, Fisher’s method, DCT, and GLCM for feature extraction and dimension reduction, and SVM, LR, RF, and k-NN for classification modeling. The deep learning strategy involved a CNN-LSTM model. In terms of data features, the most successful method was SVD. The most accurate classifier among the employed techniques was LR. Furthermore, comparison of the sensor settings demonstrated that by using only one side sensor, the most accurate results can be obtained, which simplifies the hardware and software. In addition, the generalization of the algorithms were tested in terms of deviation in layout and environmental temperature between the train and test data successfully. Regarding the layout settings, it was concluded that small layout produced better accuracy. Finally, the methodologies were also validated successfully on another dataset called *Infra-ADL2018*, including four LRIR sensors, and single-, double-, and three-subject activities.

## Figures and Tables

**Table 1:** Two categories of activities in the *Coventry-2018* dataset.

	1-Subject			2-Subject
Activities	Small	Large	Activities	Large
Sit-Down	AS1	AL1	Both Sitting	B1
Stand-Still	AS2	AL2	Sitting & Moving	B2
Sit-Down & Stand-Up	AS3	AL3	Sitting & Standing	B3
Stand-Up	AS4	AL4	Random Moving	B4
Left & Right Move	AS5	AL5	Both Standing	B5
For-backward Move	AS6	AL6	Standing & Moving	B6
Walking-Diagonally 1	AS7	AL7	Walking Across	B7
Walking-Diagonally 2	AS8	AL8		

**Table 2:** The three categories of activities of the *Infra-ADL2018* dataset.

**1-Subject**	Walking LR; Walking RL; Walking Away; Walking Toward; Falling; Stand to Sit; Sit to Stand; Sitting Still; Standing Still.
**2-Subject**	Walking Opp Direction; Walking Same Direction; Sitting; Standing; Sitting+Walking Front; Sitting+Walking Behind; Standing+Walking Front; Standing+Walking Behind; Sitting+Standing; Falling+Walking.
**3-Subject**	Free Movement; Stand Still.

**Table 3:** Testing performances for all combinations of the feature extraction and classification methods as well as CNN-LSTM using the Sensor-1 large-layout dataset from *Coventry-2018* for 15 classes of single-subject and double-subject activities.

	SVM	RF	k-NN	LR	CNN-LSTM
**SVD**	96.66 ± 0.9%	97.02 ± 1.04%	88.88 ± 1.57%	100 ± 0%	—
**Fisher**	87.03 ± 2.28%	88.14 ± 0.52%	86.29 ± 1.04%	83.33 ± 1.6%	—
**DCT**	84.95 ± 0.72%	94.1 ± 0.83%	79.97 ± 0.39%	84.36 ± 1.66%	—
**GLCM**	77.39 ± 2.09%	82.22 ± 1.81%	73.7 ± 2.09%	73.44 ± 1.91%	—
**CNN-LSTM**	—	—	—	—	95.88 ± 1.1%

**Table 4:** Testing performance of the classification models using SVD with LR classifier for different number of activities of *Infra-ADL2018*.

	Data	Single-Subject Activity	Double-Subject Activity	All Activities (21 Classes)
	
**Sensor-1**		93.44 ± 0%	100 ± 0%	93.91 ± 0.37%
**Sensor-2**		95.08 ± 0%	100 ± 0%	100 ± 0%
**Sensor-3**		100 ± 0%	100 ± 0%	97.61 ± 0%
**Sensor-4**		90.16 ± 0%	100 ± 0%	95.76± 0.37%

**Table 5:** Testing performances for all feature extraction methods and LR classifier as well as the CNN-LSTM method using the Sensor-1 large-layout dataset for 8 classes of single-subject and 7 classes of double-subject activities from the *Coventry-2018* dataset, as well as 9 classes of single-subject and 10 classes of double-subject activities from the *Infra-ADL2018* dataset.

	Coventry-2018		Infra-ADL2018	
	Single	Double	Single	Double
**SVD**	88.33 ± 0%	98.11 ± 0%	97.90 ± 1.6%	100 ± 0%
**Fisher**	86.67 ± 0%	91.82 ± 0.89%	83.05 ± 2.78%	97.21 ± 0.78%
**DCT**	78.33 ± 0%	94.33 ± 0%	89.34 ± 0.82%	100 ± 0%
**GLCM**	78.33 ± 2.71%	80.49 ± 2.35%	66.11 ± 3.36%	97.77 ± 0.78%
**CNN-LSTM**	90.74 ± 1.31%	95.83 ± 2.94%	95.13 ± 5.77%	96.66 ± 6.66%

## Data Availability

The *Coventry-2018* dataset is publicly available on IEEE DataPort: https://ieee-dataport.org/documents/infrared-human-activity-recognition-dataset-coventry-2018, 15 November 2022.
